# Sulfurization of bimetallic (Co and Fe) oxide and alloy decorated on multi-walled carbon nanotubes as efficient bifunctional electrocatalyst for water splitting

**DOI:** 10.1016/j.heliyon.2024.e32989

**Published:** 2024-06-13

**Authors:** Sana Ullah, Asif Hussain, Muhammad Asim Farid, Faiza Anjum, Roohul Amin, Shangfeng Du, Ji-Jun Zou, Zhen-Feng Huang, Muhammad Tahir

**Affiliations:** a School of Chemical Engineering and Technology, Tianjin University, Tianjin, China; b Department of Physics, University of Lahore, 53700 Lahore, Pakistan; c Department of Chemistry, University of Education Lahore, 53700 Lahore, Pakistan; d School of sciences, Tianjin University, China; e School of Chemical Engineering, Birmingham University, Birmingham, UK; f Department of Physics, University of Education, Lahore, Punjab 54770, Pakistan

**Keywords:** OER, HER, MWCNTs, Electrocatalysis, Water splitting, Bifunctional electrocatalyst

## Abstract

The advancement in electrocatalysis, particularly in the development of efficient catalysts for hydrogen and oxygen evolution reactions (HER and OER), is crucial for sustainable energy generation through processes like overall water splitting. A notable bifunctional electrocatalyst, CoFe_2_O_4_/Co_7_Fe_3_, has been engineered to facilitate both OER and HER concurrently, aiming to reduce overpotentials. In the pursuit of further enhancing catalytic efficiency, a morphological transformation has been achieved by introducing a sulphur source and multi-walled carbon nanotubes (MWCNTs) into the catalyst system, resulting in S–CoFe_2_O_4_/Co_7_Fe_3_/MWCNTs. This modification has significantly improved the activity for both OER and HER. An onset overpotential of 250 mV@10 mAcm^−2^ for the OER and 270 mV@50 mAcm^−2^ for the HER, indicating efficient catalytic activity at relatively low overpotentials. S–CoFe_2_O_4_/Co_7_Fe_3_/MWCNTs display an outstanding long-term stability in alkaline electrolytes, with minimal Tafel slopes of 77 mV/dec for the OER and 70 mV/dec for the HER, suggesting sustained catalytic performance over extended periods. Furthermore, when employed as both the cathode and anode in the context of complete water splitting, S–CoFe_2_O_4_/Co_7_Fe_3_/MWCNTs demonstrate an impressive cell voltage of 1.52 V at a current density of 10 mA cm^−2^ in a 1 M KOH solution, showcasing its viability for practical applications. Given its cost-effectiveness and superior activity, S–CoFe_2_O_4_/Co_7_Fe_3_/MWCNTs hold significant promise for widespread applications in overall water splitting electrocatalysis, contributing to the advancement of cleaner and sustainable fuel generation technologies.

## 1 Introduction

The production of oxygen and hydrogen from overall water splitting provides an eco-friendly method for the generation and storage of renewable energy, to overcome on the energy shortage for sustainable environment [[1], [2], [3]]. Subsequently, extensive research has been conducted to introduce catalysts with desirable properties and stability [4,5].

The water splitting process has two types of half-reaction, oxygen evolution reaction (OER) and hydrogen evolution reaction (HER) which were discovered in 1789. The quantitative relationship between electrical energy consumed and the gas produced was established using Faraday's law in 1833. Dmitry Lachinov used alkaline water electrolysis for hydrogen production at industrial scale in 1888 [6]. In the early 20th century the excellent OER and HER materials were Ni-based oxides and Ni-based alloys [7]. The fundamental requirements for water splitting are lower overpotential and higher stability of electrocatalysts [8,9]. In this regard, rare metal-based catalysts like IrO_2_, RuO_2_ and Pt processing high potential to produce oxygen and hydrogen [10]. However, the high price and scarcity of abovementioned precious metals make their commercial use unattractive [11,12]. Thus, numerous efforts have been made for the exploration of stable non-precious metal-based water splitting electrocatalysts, such as carbides, chalcogenides, transition metal sulfide, transition metal phosphide, transition metal oxides, bimetallenes, hydroxides and metal organic frameworks [[13], [14], [15], [16], [17], [18], [19]].

The diversified nanostructures and excellent properties make oxides of transition metals an excellent and progressive electrocatalysts [[20], [21], [22], [23]]. Such transition metal oxides, especially metals ferrite M − Fe_2_O_4_ (M = Ni, Co, and Cu) have emerged as an essential and high potential electrocatalysts for water splitting. The reasons for their recommendation are their outstanding activities, long-term stability, and environmental friendliness [[24], [25], [26], [27]]. Since M − Fe_2_Co_4_ can indeed exhibits certain challenges as an electrocatalyst, undergo slow reaction kinetics, limited active sites and degradation or structural changes over extended cycles of HER and OER [28] The alloys of transition metals (FeCo, CoNi, and FeNi) have greater performance compared with their single metallic constituents [29]. Moreover, M − Fe_2_Co_4_ can indeed exhibits certain challenges as an electrocatalyst, undergo slow reaction kinetics, limited active sites and degradation or structural changes over extended cycles of HER and OER. Additionally, metallic alloys play an active role in various electronic structures, contributing to the superior performance of electrocatalysts [30,31]. For instance, Bao's research team synthesized binary transition-metal alloys, including elements such as Co, Ni, and Fe. Through optimization of the FeNi composition, they successfully developed a high-performance oxygen evolution reaction (OER) catalyst exhibiting the best activity and superior durability [32,33], which can be enhanced by combining them with other conductive materials, like graphene and multiwall carbon nanotubes (MWCNTs). Moreover, the improvement of catalytic activities of spinal ferrites (CoFe_2_O_4_/Co_7_Fe_3_) has been achieved by incorporating anions, such as sulphur anions, which are valuable for the increase of catalytic sites [[34], [35], [36]] Sun et al. reported the incorporation of sulphur in CoFe_2_O_4_/MWCNTs, resulting in higher oxygen evolution reaction (OER) performance compared to the catalyst without sulphur incorporation [[37], [38], [39]].

In this research work, we studied the sulfurization of cobalt iron oxide (CoFe_2_O_4_) and an alloy of cobalt iron (Co_7_Fe_3_) decorated on MWCNTs, (S–CoFe_2_O_4_/Co_7_Fe_3_/MWCNTs) for overall water splitting. The experimental results showed that the synthesized electrocatalyst (S–CoFe_2_O_4_/Co_7_Fe_3_/MWCNTs) is highly capable for HER and OER due to their unique morphology and composition. The OER and HER activities of our new catalyst are superior to the same performance over a noble and precious metal-based catalyst such as IrO_2_, RuO_2_, and Pt/C.

## 2 Experimental section

### 2.1 Reagents used for the synthesis of catalyst

The chemicals used for the synthesis of catalysts such as CoCl_2_.6H_2_O, FeCl_3_.4H_2_O, Urea, Thiourea, MWCNTs, Pt/C, IrO_2_ and RuO_2_ were purchased from J & K chemicals. An absolute ethanol was bought from Tianjin Guangfu fine chemical research Centre and 5 percent Nafion solution is purchased from Sigma Aldrich. Highly pure distilled water was gotten from the UP-water purification system based in Tianjin.

### 2.2 Synthesis of CoFe_2_O_4_/Co_7_Fe_3_

The nanoparticles of cobalt iron oxide with cobalt iron alloy (CoFe_2_O_4_/Co_7_Fe_3_) were synthesized through a one-step process. 0.0019 M (0.7 mg) of CoCl_2_·6H_2_O, 90.006 M (1.5 mg) of FeCl_3_·4H_2_O and 0.03 M (1.8 mg) of urea were dissolved in 20 ml of deionized water. All required chemicals were mixed and magnetically stirred for 30 min. The pH value of the solution was increased to 12.56 by the addition of NaOH during stirring. Then the attained mixture was shifted to a 100 ml Teflon-lined stainless-steel autoclave and heated up at 200 °C for a 24 h. Later, the autoclave was cooled down to room temperature. The black nanoparticles were cleaned with water and ethanol several times and collected by centrifugation. The obtained nanoparticles were dried at 80 °C for 12 h fallowed by annealing step at 800 °C for 2 h in an argon atmosphere. The catalytic material, cobalt iron oxide with cobalt iron alloy (CoFe_2_O_4_/Co_7_Fe_3_) was obtained when the tube furnace was cooled down under normal conditions.

### 2.3 Synthesis of S–CoFe_2_O_4_/Co_7_Fe_3_ and S–CoFe_2_O_4_/Co_7_Fe_3_/MWCNTs

To synthesize S–CoFe_2_O_4_/Co_7_Fe_3_, 0.5 g of CoFe_2_O_4_/Co_7_Fe_3_ and 0.3 g of thiourea were physically mixed and dried at 60 °C for 12 h. The obtained sample was heated at 300 °C for 2 h in the presence of an argon atmosphere. A similar process was also used for the synthesis of S–CoFe_2_O_4_/Co_7_Fe_3_/MWCNTs where additionally 4 ml of MWCNTs were added along with other reactants.

### 2.4 Characterizations

X-ray diffraction (XRD) patterns of the prepared samples were recorded by a Bruker D8 focus operating at 45 kV and 45 mA furnished with nickel-filtered Cu *Kα* (λ = 0.15405 nm) radiation in the range of 10°–80° of 2θ to find the information about structures. Elemental mapping of bimetallic oxide and alloy (S–CoFe_2_O_4_/Co_7_Fe_3_), decorated on MWCNTs was studied using an energy dispersive X-ray spectroscopy (EDX) coupled with field-emission scanning electron microscope (Hitachi S-4800). The morphological analysis was carried using tecnai G2 F-20 transmission electron microscope. Chemical state of the samples were analysed with a PHI-5000 versa probe X-ray photoelectron spectroscope (XPS) with Al *Kα* radiation having energy 1486.6 eV. Electron binding energies were calibrated against the C 1s emission at *E*_b_ = 284.8 eV. Surface area was investigated by using Brunauer Emmett-Teller (BET) instrument ST 3H-2000 PS2 through the BET method.

### 2.5 Electrochemical measurements

Nickel foam was washed with dilute HCl and dried at 60 °C for 12 h. The homogeneous mixture of ink was loaded using micropipette. When the ink was completely loaded to Ni foam it was then dried at room temperature. Activity of this newly electrocatalyst is checked by electrochemical workstation. Electrochemical measurements were performed using IVIUMSTAT workstation (IVIUM Technologies BV, Netherlands). This workstation consisted of three types of electrodes, a working electrode (catalyst on Ni-foam), graphite rod used as counter electrode and reference electrode made as Hg/HgO. 5 mg of the prepared catalyst was added to 1 ml of ethanol (analytical grade 99 %) along with 20–30 μL of Nafion binder where the solution was then sonicated for 10 min. The prepared ink was loaded to Ni-Foam by a micropipette and dried at 60 °C. All the experiments were carried out in 1 M KOH electrolytes with a scan rate of 5 mV s^−1^. Linear sweep voltammetry (LSV) and all electrode potentials were converted to a reversible hydrogen electrode. Moreover, the active surface area of the electrocatalyst is attained from double-layer capacitance of 10–100 mV s^−1^. A reversible hydrogen electrode (RHE) is used for potential reference and calculated using Equation (1) as follows:

(1) $$E\left(\text{RHE}\right)=E\left(\frac{H_{g}}{H_{g}}O\;1\;M\;\text{KOH}\right)+0.059\;\text{pH}+0.133V$$

Two sets of electrodes; anode (S–CoFe_2_O_4_/CO_7_Fe_3_/MWCNTs), and cathode (S–CoFe_2_O_4_/Co_7_Fe_3_/MWCNTs) were used for overall water splitting in 1 M KOH electrolytes. pH of the solution (12.5) was measured using pH meter and the scan rate of linear sweep voltammetry (LSV) was set to 5 mV s^−1^.

### 2.6 Parameter for catalytic performance Calculation

Tafel slope analysis is employed to diagnose the reaction mechanism, kinetics, and compare the different catalytic activities. The rate-determining step is dependent on the Tafel slope, calculated using Equation (2) given below.

(2) $$\eta =b\;\log\left(\frac{J}{J_{o}}\right)$$

where, *η* denotes the overpotential, *b* denotes the Tafel slope, *J* represented current density, and *J*_*o*_ is used for the exchange of current density. The Low value of Tafel plot was the representation of good OER activity.

Turnover frequency (TOF) was intended from Equation (3) which was given below.

(3) TOF=J×S/4×f×n

where *J* denoted the current density in (mA.cm^−2^) at a definite voltage, *S* stands for the geometric area of the electrode, *n* is the number of moles of the electrocatalyst understudy and “*f”* is used for faraday constant (96485.3C mol^−1^)

The cyclic Voltagrams (CVs) were measured at different scan rates, CV represented the current density versus the scan rate for S–CoFe_2_O_4_/MWCNTs. The electrochemically active surface area (ECSA) is calculated by using Equation (4) given below:

(4) $$ESCA=\frac{C_{dl}}{CVs}$$

the c_dl_ of (S– CoFe_2_O_4_/Co_7_Fe_3_/MWCNTs) is 51.0 mF cm^−2^, which was more significant than the cdl value individually measured for CoFe_2_O_4_/Co_7_Fe_3_, S–CoFe_2_O_4_/Co_7_Fe_3_ and CoFe_2_O_4_/Co_7_Fe_3_/MWCNTs. The chronopotentiometry was used to check the stability of the synthesized catalysts.

Electrical impendence spectroscopy (EIS) was estimated from Nyquist plots, which are data plots for numerous frequencies as a set of points in the complex impedance plane taken as Zʹ vs Zʺ. These plots measure equivalent series resistance (Rs), which is used for the detection of charge transfer resistance (Rct) which reveals the ohmic resistance (electrical conductivity) useful for the study of kinetics in catalyst samples. The electrical impendence spectroscopic experiments were measured from 100,000–0.1 Hz with 10 mV amplitude, where the Rct follows the same order for all samples.

## 3 result and discussion

### 3.1 Physicochemical characterization of S–CoFe_2_O_4_/Co_7_Fe_3_/MWCNTs

Structural and morphological characterization of as-synthesized product (S–CoFe_2_O_4_/Co_7_Fe_3_/MWCNTs) was done by using XRD, field-emission scanning electron microscopy (FESEM), and TEM.

**Fig. 1:**
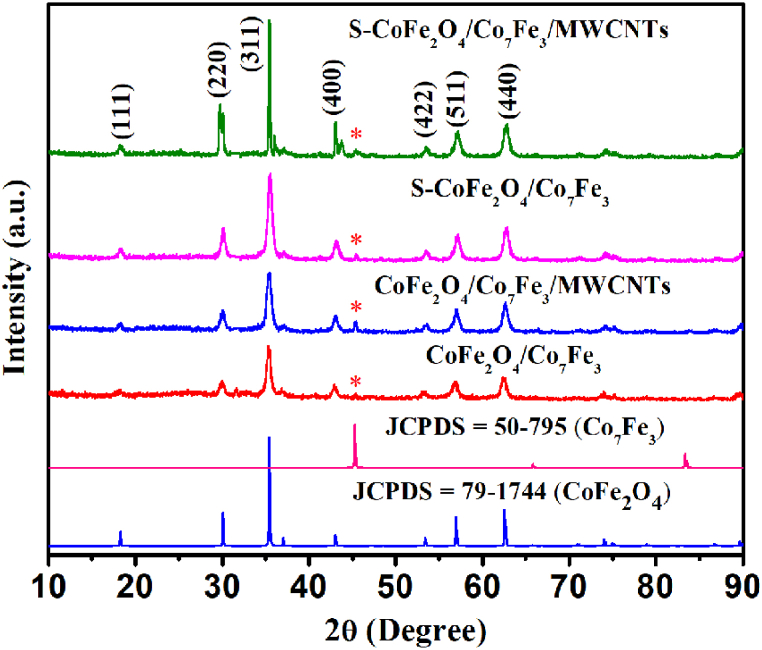
XRD pattern of S–CoFe_2_O_4_/Co_7_Fe_3_/MWCNTs.

Fig. 1 represents XRD patterns of synthesized catalysts CoFe_2_O_4_/Co_7_Fe_3_), CoFe_2_O_4_/Co_7_Fe_3_/MWCNTs, S–CoFe_2_O_4_/Co_7_Fe_3_ and S–CoFe_2_O_4_/Co_7_Fe_3_/MWCNTs. The XRD patterns for bimetallic oxide was matched with standard ICDD/PDF No. 01-79-1744 for CoFe_2_O_4_, and for the bimetallic alloy it was matched with standard card of ICDD/PDF No. 00-050-0795 for Co_7_Fe_3_. The XRD short peak at 45° marked with artistic denoted the presence of Co_7_Fe_3_ [40].

**Fig. 2:**
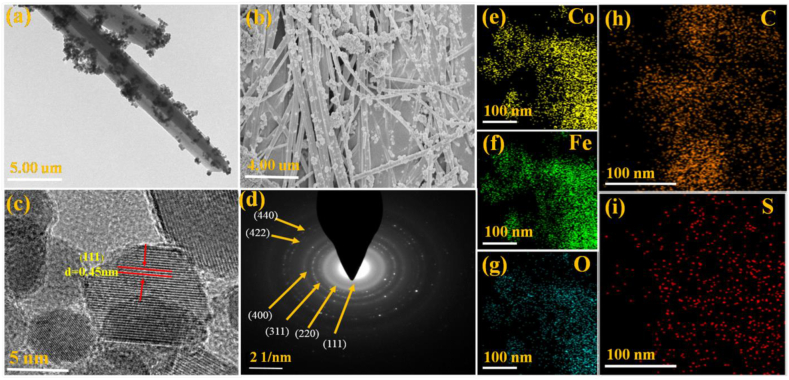
TEM images of S–CoFe_2_O_4_/Co_7_Fe_3_/MWCNTs at 5.00 μm (a) TEM of S–CoFe_2_O_4_/Co_7_Fe_3_/MWCNTs at 4.00 μm (b) HRTEM of S– CoFe_2_O_4_/Co_7_Fe_3_ MWCNTs at 4.00 μm (c) SAED Pattern (d) Element mapping of Co (e), Fe (f), O (g), C (h), and S (i) in S–CoFe_2_O_4_/Co_7_Fe_3_/MWCNTs.

In Fig. 2(a and b), transmission electron microscopy (TEM) images of S–CoFe_2_O_4_/Co_7_Fe_3_/multi-walled carbon nanotubes (MWCNTs) at 5.00 μm and 4.00 μm are depicted. These images reveal the distinctive morphology of synthesized catalyst, a crucial factor contributing to its enhanced performance in alkaline electrolytes for overall water splitting. The lattice spacing measurements of 0.45 nm and 0.35 nm, corresponding to the (111) and (220) planes of S–CoFe_2_O_4_/Co_7_Fe_3_/MWCNTs, respectively, are shown in Fig. 2(c and d). This information underscores the structural characteristics of the catalyst, indicating its potential for efficient water splitting in alkaline environments. This Figure also represented the SAED pattern (scattered area electron diffraction) which further confirmed high crystallinity of newly synthesized catalyst. It is confirmed from the SAED, which is perfect concentric diffraction pattern and corresponding to the (111), (220), (311), (400), (422), (511) and (440) crystal planes of CoFe_2_O_4_/Co_7_Fe_3_ and well consistent with XRD pattern.

Elemental percentage of (Co, Fe, O, C, S) in S–CoFe_2_O_4_/Co_7_Fe_3_/MWCNTs catalysts is shown in Fig. 2(e-i). The EDX and element mapping denotes the distribution of Fe, Co, S, C, and O along with molar contents of Co, Fe, C, S, and O which is approximately 14.24, 27.98, 12.71, 6.58, and 38.49 % respectively as listed in Table 1. Thus, the presence of sulphur and CoFe_2_O_4_/Co_7_Fe_3_ on the surface of MWCNTs is confirmed. The above characterization clearly shows that cobalt iron oxide with cobalt iron (CoFe_2_O_4_/Co_7_Fe_3_) nanoparticle having cubic crystal structure has been synthesized successfully. Further, it is also confirmed that sulphur and CoFe_2_O_4_/Co_7_Fe_3_ are well decorated on the surface of MWCNTs.

**Table 1:** Elemental percentage of (Co, Fe, O, C, S) in S–CoFe_2_O_4_/Co_7_Fe_3_/MWCNTs catalyst.

Element	Weight %	Atomic %
C K	12.71	24
O K	38.49	54.51
S K	6.58	4.66
Fe K	27.98	11.35
Co K	14.24	5.48

**Fig. 3:**
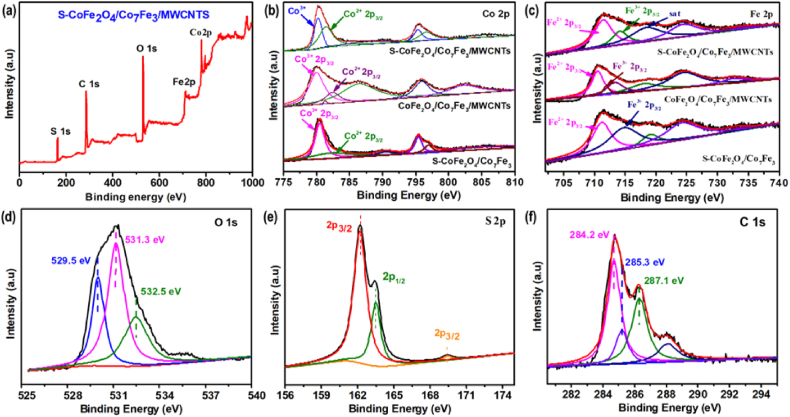
XPS spectra of **(a)** catalyst S–CoFe_2_O_4_/Co_7_Fe_3_/MWCNTs, **(b)** Co 2p core levels and **(c)** Fe 2p core levels of (S–CoFe_2_O_4_/Co_7_Fe_3_, CoFe_2_O_4_/Co_7_Fe_3_/MWCNTs and S–CoFe_2_O_4_/Co_7_Fe_3_/MWCNTs), **(d)** O 1s core levels, **(e)** S 2p core levels and **(f)** C ls core levels of S–CoFe_2_O_4_/Co_7_Fe_3_/MWCNTs.

XPS measurements were recorded to investigate the chemical nature and chemical bonding of sulfurization of cobalt iron oxide and alloy (S–CoFe_2_O_4_/Co_7_Fe_3_) decorated on MWCNTs. Fig. 3(a) represents complete scan spectra of XPS showing the presence of Co, Fe, C, S and O in S–CoFe_2_O_4_/Co_7_Fe_3_/MWCNTs. The existence of these elements exhibits strong agreement with elemental mapping. Furthermore, Fig. 3(b) represents two main peaks for Co 2p core level located at 780.31 eV and 795.33 eV for all samples of S–CoFe_2_O_4_/Co_7_Fe_3_/MWCNTs and S–CoFe_2_O_4_/Co_7_Fe_3_. The positions of Co 2p_3/2_ and Co 2p_1/2_ are also mentioned in the catalyst CoFe_2_O_4_/Co_7_Fe_3_/MWCNTs representing a unique pattern, whereas the peak width is even higher than other samples. There are two core peaks observed at 780.33 eV and 795.33 eV while, corresponding satellite peaks are located at 786.1 eV and 802.8 eV. These satellite peaks also represents the presence of Co^2+^ state [41,42]. The small peak at 781.27 eV denoted that Co 2p shifted to lower binding energy when cobalt is doped on another anion catalyst [43,44]. Fig. 3 (c) represents the comparison of Fe 2p for all samples containing two main peaks in the range of 711.53 eV (2p_2/3_) and other at 724.5 eV (2p_1/2_). Meanwhile, two satellite peaks are also shown at the range of 718.8 eV. Only CoFe_2_O_4_/Co_7_Fe_3_ indicates another satellite peak at the 732.4 eV while, this satellite peak is missing in S–CoFe_2_O_4_/Co_7_Fe_3_ catalyst. In the case of Fe 2p spectrum, the peak at 718.8 eV represents the Fe^3+^ oxidation state of iron [45,46]. Furthermore, two peaks of S 2p_1/2_ and 2p_3/2_ are shown at 162 eV and 163 eV confirmed the addition of sulphur and another peak at 169.52 eV showed sulphur oxide. The presence of the S 2p peak is evidence that sulphur anion is completely doped on CoFe_2_O_4_/Co_7_Fe_3_ [41,47].

Similarly, C 1s spectra indicates three absorbance peaks for oxygenated carbon at 284.2 eV, 286 eV and 287.1 eV which are contributed by C–O and C=O respectively [48], another small peak at 285.1 eV represents C=C. Moreover, the peak at 284.0 eV low binding energy is possible to show the presence of bonding of carbon with metals oxide [48]. Fig. 3(d) represents the O 1s spectrum, which includes three main peaks at 529.5 eV, 531.3 eV and 532.5 eV, the peak at 531.3 eV is more abundant than the other two peaks. This peak is associated with surface hydroxyl species and a smaller peak at 529.5 eV is associated with sulphide species [41,49].

### 3.2 Electrocatalytic OER performance in alkaline media

**Fig. 4:**
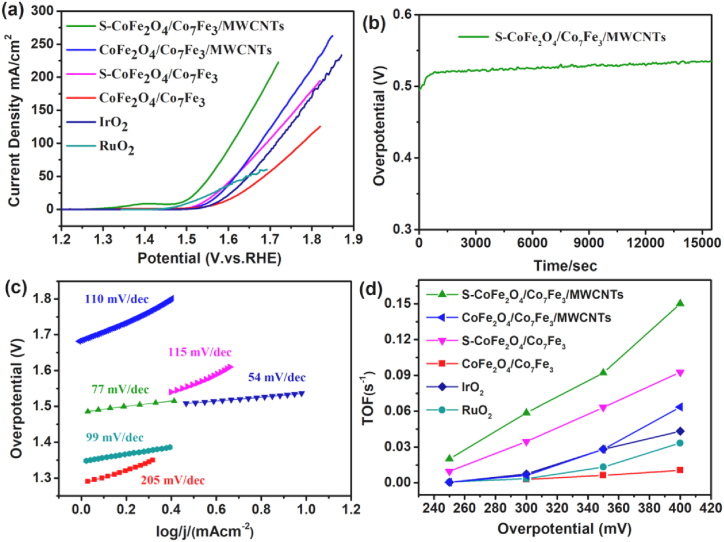
LSV of OER (a), OER stability (b), Tafel slope (c) and TOFs for OER (d).

We studied the sulfurization of bimetallic oxide and alloy (S–CoFe_2_O_4_/Co_7_Fe_3_) decorated on MWCNTs for OER and HER in 1 M KOH solution and its further application for water splitting. Initially, the synthesized samples are investigated for OER on Ni-foam, the OER polarization curves which are noted with a lower scan rate (5 mVs^−1^). Fig. 4(a) shows the LSV curves of CoFe_2_O_4_/Co_7_Fe_3_, S–CoFe_2_O_4_/Co_7_Fe_3_/MWCNTs, S–CoFe_2_O_4_/Co_7_Fe_3_, CoFe_2_O_4_/Co_7_Fe_3_/MWCNTs, IrO_2_, and RuO_2_ respectively in alkaline media. The current density is measured at 10 mAcm^−2^. The new synthesized catalyst (S–CoFe_2_O_4_/Co_7_Fe_3_/MWCNTs) exhibited better performance and showed that overpotential of 250 mV versus RHE is required to achieve current density of 10 mAcm^−2^. Moreover, the activity of S–CoFe_2_O_4_/Co_7_Fe_3_/MWCNTs is better than other synthesized materials and better than IrO_2_ (310 mV at 10 mAcm^−2^). This indicates that the synthesized hybrid catalyst, through sulfurization of bimetallic oxide and alloy (S–CoFe_2_O_4_/Co_7_Fe_3_) decorated on MWCNTs with low potential are more useful than a noble metal-based commercial electrocatalyst. Meanwhile, outstanding stability is another critical factor for the catalytic activity of the catalyst. The OER stability for our best catalyst (S–CoFe_2_O_4_/Co_7_Fe_3_/MWCNTs) for 250 h is represented in Fig. 4(b), which shows that at a constant current range as no variation is observed during this period. Furthermore, to explore the catalytic kinetics for OER, a Tafel plot is obtained which demonstrates that how current is responsive to overpotential. Fig. 4(c) describes the comparison of the Tafel slope values for all the samples. Tafel slope for the best sample, sulfurized cobalt iron oxide and alloy (S–CoFe_2_O_4_/Co_7_Fe_3_) decorated on MWCNTs, (S–CoFe_2_O_4_/Co_7_Fe_3_/MWCNTs) was measured to be 77 mV/dec, which is comparable to the state-of-the-art values of Tafel slope of IrO_2_ and RuO_2_ which is 54 mV/dec and 99 mV/dec, respectively.

The small value of the Tafel slope for S–CoFe_2_O_4_/Co_7_Fe_3_/MWCNTs also confirmed that low overpotential will be required to support better OER since a value of Tafel slope less than 120 mV/dec of is more favourable for OER [50]. The turnover frequency (TOF) is another essential factor in evaluating the better performance of OER [14,51]. The TOF of S–CoFe_2_O_4_/Co_7_Fe_3_ decorated on MWCNTs is measured to be 0.15 (s^−1^) at a potential of 250 mV, which is found to be greater than RuO_2_ and IrO_2_. The higher TOF value of S–CoFe_2_O_4_/Co_7_Fe_3_/MWCNTs as shown in Fig. 4(d) is also evidence for the better performance of OER. The process mechanism of OER can be explained through four steps in alkaline medium: (1) formation of adsorbed M-OH* on the catalysts by the reaction (M + OH^−^ → MOH*), (2) transformation of MOH* to MO* by the reaction (MOH* + OH^−^ → MO* + H_2_O), (3) transformation of M-O* to MOOH* through the reaction (MO + OH^−^ → MOOH*), and (4) release of O_2_ by the reaction (MOOH* + OH^−^ → M + O_2_ + H_2_O + e^−^). Since the S–CoFe_2_O_4_/Co_7_Fe_3_/MWCNTs contains iron and cobalt metals which make more stable electrocatalyst to perform above mentioned four steps of OER.

**Fig. 5:**
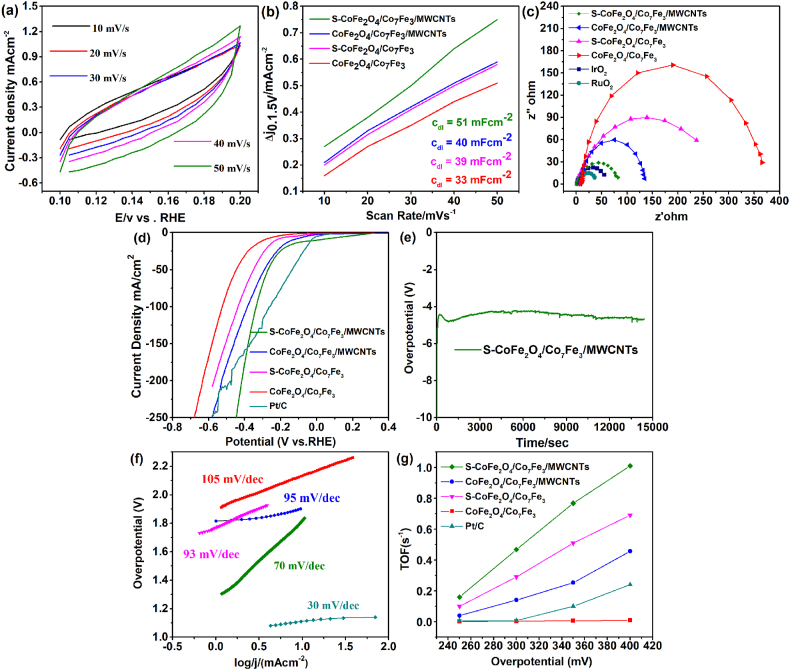
Cyclic voltammetry (CV) (a), double-layer capacitance (C_dl_) (b) and electrochemical impedance (EIS) of S–CoFe_2_O_4_/Co_7_Fe_3_/MWCNTs (c) LSV curves (d) stability test (e) Tafel slope (f) and TOFs (g) of HER for different samples.

The cyclic Voltagrams (CV) are exhibited as another factor at various scan rates (10–100 mV/s, as given in Fig. 5(a). Each CV curve represented a leaf like shape on every scan rate, which enhanced the double-layer capacitance (C_dl_). The C_dl_ is directly associated with the number of the active sites of the catalyst and it also normalized the exchange's current density, which revealed more catalytic activity [52]. It is worth noticing that our best sample (S–CoFe_2_O_4_/Co_7_Fe_3_/MWCNTs) has C_dl_ value of 51.0 mF cm^−2^, which is significantly greater than values recorded by all other synthesized samples as shown in Fig. 5(b). The higher current density of S–CoFe_2_O_4_/Co_7_Fe_3_/MWCNTs is due to the addition of sulphur, whereas the addition of MWCNTs resulted in more active sites. Furthermore, the outstanding performance of S–CoFe_2_O_4_/Co_7_Fe_3_/MWCNTs is exhibited by its stability which is another essential influence that verified the catalytic activity of the synthesized catalyst.

The catalyst S–CoFe_2_O_4_/Co_7_Fe_3_/MWCNTs provided better stability in the alkaline electrolyte and no significant changes are noted during this period. The electrochemical impedance (EIS) is another critical factor for the analysis of kinetics through the catalytic process, from the EIS study charge transfer resistance is determined. The smaller semicircle of EIS is often related to the fast electron transmission process. The cobalt iron oxide (CoFe_2_O_4_) and alloy of cobalt iron (Co_7_Fe_3_) exhibit a large semicircle and showed higher charge transfer resistance of about 350 Ω. Whereas, with the addition of sulphur and MWCNTs the resistance of S–CoFe_2_O_4_/Co_7_Fe_3_/MWCNTs is near to 83 Ω. At the same time, the addition of sulphur and MWCNTs to CoFe_2_O_4_/Co_7_Fe_3_, promote better electron transmission across the catalytic process.

From Fig. 5(c) it is noteworthy that S–CoFe_2_O_4_/Co_7_Fe_3_/MWCNTs show resistance of 83 Ω, which has smaller Rct than S–CoFe_2_O_4_/Co_7_Fe_3_ and CoFe_2_O_4_/Co_7_Fe_3_/MWCNTs. Moreover, the electrical conductivity and the number of active sites are essential factors for the better performance of an electrocatalyst [53,54]. The increase in the conductivity as well as enhancement of the active sites of catalysts leads to progressive activities. The sulphur and MWCNTs are acting as a conducting background for faster electron transmission thus leading to better catalytic property for overall water splitting. Therefore, the newly synthesized catalyst sulfurized with cobalt iron oxide (CoFe_2_O_4_) and alloy of cobalt iron (Co_7_Fe_3_) decorated on MWCNTs, (S–CoFe_2_O_4_/Co_7_Fe_3_/MWCNTs) showed better performance.

### 3.3 Electrocatalytic HER performances in alkaline media

Hydrogen evolution reaction (HER) performance of the sulfurized cobalt iron oxide (CoFe_2_O_4_) and alloy of cobalt iron (Co_7_Fe_3_) decorated on MWCNTs, formed S–CoFe_2_O_4_/Co_7_Fe_3_/MWCNTs is studied in alkaline media and Hg/HgO is used as a reference electrode. Fig. 5(d) represents the HER polarization curves for all synthesized samples. Our best sample (S–CoFe_2_O_4_/Co_7_Fe_3_/MWCNTs) has a small potential of 270 mV at 50 mAcm^−2^ for HER. The results of HER activities are matched with the commercial catalyst such as Pt/C. It was observed that the performance of CoFe_2_O_4_/Co_7_Fe_3_ itself as well as on Ni-foam was inferior. However, the addition of sulphur and MWCNTs to CoFe_2_O_4_/Co_7_Fe_3_ suddenly increased the activities of (S–CoFe_2_O_4_/Co_7_Fe_3_/MWCNTs). Moreover, transition metal alloy has a crucial role in the enhancement of hydrogen evolution reaction. Meanwhile the HER stability for our best catalyst (S–CoFe_2_O_4_/Co_7_Fe_3_/MWCNTs) is represented Fig. 5 (e) which, shows that at a constant current range as no variation is observed during this period. To explore the catalytic kinetics for OER, a Tafel plot is obtained which demonstrates that how current is responsive to overpotential. Fig. 5(f) describes the comparison of the Tafel slope values for all the samples. Tafel slope for the best sample, sulfurized cobalt iron oxide and alloy (S–CoFe_2_O_4_/Co_7_Fe_3_) decorated on MWCNTs, (S–CoFe_2_O_4_/Co_7_Fe_3_/MWCNTs) was measured to be 70 mV/dec, which is comparable to the state-of-the-art values of Tafel slope of Pt/C which is 30 mV/dec. Furthermore, as shown in Fig. 5(g), the turnover frequency (TOF) measurement for S–CoFe_2_O_4_/Co_7_Fe_3_/MWCNTs is 1.01 (s^−1^) at the overpotential of 40 mV at 10 mA cm^−2^, which is much better than (TOF) values for S–CoFe_2_O_4_/Co_7_Fe_3_ and CoFe_2_O_4_/Co_7_Fe_3_/MWCNTs.

The reaction mechanism of the HER in alkaline medium can be understood through three fundamental steps: (1) H_2_O + M + e^−^ ⇆ M-H_ad_ + OH^−^ (the Volmer step), (2) H_2_O + M-H_ad_ + e^−^ ⇆ M + H_2_ + OH^−^ (the Heyrovsky step) and (3) 2M-H_ad_ ⇆ 2 M + H_2_ (the Tafel recombination step). It is expected that the unique morphology of transition metal alloy, the addition of sulphur as an active metal, and MWCNTs have collectively enhanced the performance of the catalyst. Moreover, sulphur and MWCNTs must be able to make a bond with another compound [55,56]. It is expected that valence shell electrons of sulphur are incomplete, and sulphur need to complete the valence shell and must make a bond with another compound. The presence of partially negative and partially positive ions helps to absorb the water molecules on the catalyst surface, which is an important step for OER and HER. Furthermore, a better synergistic relationship between sulphur and cobalt iron oxide (CoFe_2_O_4_) and alloy of cobalt iron (Co_7_Fe_3_) decorated on MWCNTs with a unique cylindrical structure made the catalyst better for water splitting. Whereas the active surface area, and easily existing redox sites are also expected that catalyst exhibit an excellent performance for HER and OER. Thus S–CoFe_2_O_4_/Co_7_Fe_3_/MWCNTs is a novel bifunctional electrocatalyst for energy conversion based on the earth-abundant metal electrocatalyst.

### 3.4 Electrocatalytic water splitting

The above results showed that sulfurization of cobalt iron oxide (CoFe_2_O_4_) and alloy of cobalt iron (Co_7_Fe_3_) decorated on MWCNTs, (S–CoFe_2_O_4_/Co_7_Fe_3_/MWCNTs) exhibited outstanding performance for HER and OER in alkaline electrolytes.

**Fig. 6:**
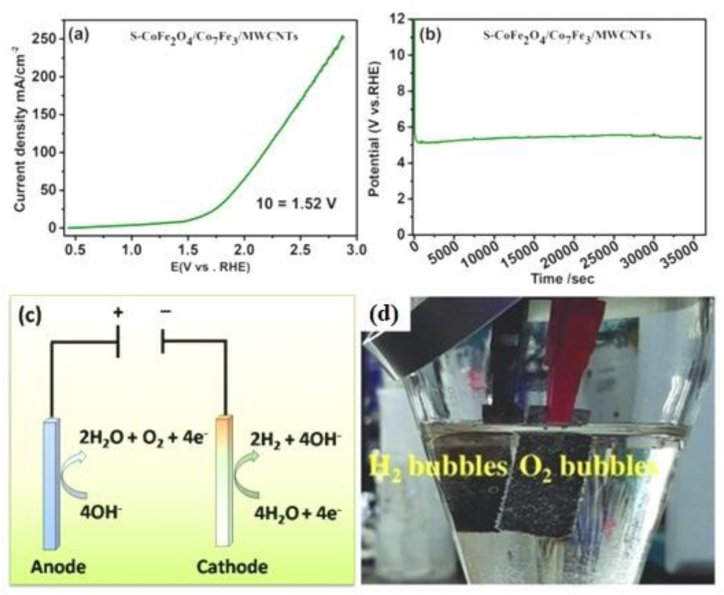
(a) Polarization curve for overall water splitting in alkaline media, (b) stability of two electrodes, (c) schematic diagram of the electrochemical cell for water splitting and (d) optical image of the electrochemical cell.

It is predictable that (S–CoFe_2_O_4_/Co_7_Fe_3_/MWCNTs) would be a suitable bifunctional electrocatalyst for overall water splitting Fig. 6(a). Therefore, two pieces of Ni-foam containing the catalyst are directly used as anode and cathode to expose their potential for water splitting. Notably, at the current density of 10 mA cm^−2^, the cell voltage of 1.52 V is achieved which is better than results of Pt/C, RuO_2,_ and IrO_2_. To the best of our knowledge, the sulfurization of cobalt iron oxide (CoFe_2_O_4_) and alloy of cobalt iron (Co_7_Fe_3_) decorated on MWCNTs (S–CoFe_2_O_4_/Co_7_Fe_3_/MWCNTs is an active and highly stable candidate for overall water splitting. It is noteworthy that durability of sulfurization of S–CoFe_2_O_4_/Co_7_Fe_3_/MWCNTs is studied at 10 mA cm^−2^ for 35000 min and only a negligible increase in the overpotential is observed after a long time as shown in Fig. 6(b). The schematic and optical image of electrocatalytic water splitting is shown in Fig. 6(c and d) Initially. S–CoFe_2_O_4_/Co_7_Fe_3_/MWCNTs are used for overall water splitting, having greater current density. This may be due to the new environment of double electrode, however, after 30 min it displayed a stable potential with 1.52 V at 10 mAcm^−2^. The outstanding catalytic activity and better stability of (S–CoFe_2_O_4_/Co_7_Fe_3_/MWCNTs) on Ni-Foam are based on the following illustrious factors, sulphur and sulphur binary compound are highly reactive, and similarly, metallic oxide and binary oxide are extremely responsive towards OER [57]. Transition metal alloy is also reactive towards HER activities, here in our catalyst cobalt iron oxide (CoFe_2_O_4_) and alloy of cobalt iron (Co_7_Fe_3_) with a unique morphological form, is also suitable candidate for better activity for HER [57,58]. The porous morphology of the Ni-foam electrode not only confirms easy contact of the surface-active sites with the electrolyte but also improved the OER and HER activities.

## 4 Conclusion

Sulfurization of cobalt iron oxide (CoFe_2_O_4_) and alloy of cobalt iron (Co_7_Fe_3_) decorated on MWCNTs resulted in (S–CoFe_2_O_4_/Co_7_Fe_3_/MWCNTs) is a promising bifunctional electrocatalyst to replace noble metal electrocatalyst for overall water splitting. S–CoFe_2_O_4_/Co_7_Fe_3_/MWCNTs showed an overpotential of 40 mV and 250 mV for HER and OER respectively at 10 mA cm^−2^ current density in 1 M KOH aqueous solution with a minimal Tafel slope of 70 mV/dec for the HER and 77 mV/dec for the OER. Notably, when sulfurized cobalt iron oxide (CoFe_2_O_4_) and alloy of cobalt iron (Co_7_Fe_3_) decorated on MWCNTs, (S–CoFe_2_O_4_/Co_7_Fe_3_/MWCNTs) is applied for the overall water electrolysis catalyst, it shows 1.52 V at 10 mA cm^−2^ and no change is observed in basic media. The long-term stability of S–CoFe_2_O_4_/Co_7_Fe_3_/MWCNTs in alkaline electrolyte is confirmed at 10 mA cm^−2^ for 35000 min where only a negligible increase in the overpotential is observed after a long time. Moreover, this work not only provides a stable and an efficient low-cost bifunctional electrocatalyst for overall water splitting but also provides a secure and easy method to realize the activities of electrocatalysts by increasing their conductivities ultimately.

## CRediT authorship contribution statement

**Sana Ullah:** Writing – original draft. **Asif Hussain:** Writing – review & editing. **Muhammad Asim Farid:** Writing – review & editing. **Faiza Anjum:** Methodology. **Roohul Amin:** Validation, Methodology. **Shangfeng Du:** Writing – review & editing, Methodology. **Ji-Jun Zou:** Supervision, Methodology. **Zhen-Feng Huang:** Supervision. **Muhammad Tahir:** Writing – review & editing, Supervision, Conceptualization.

## Declaration of competing interest

The authors declare that they have no known competing financial interests or personal relationships that could have appeared to influence the work reported in this paper.
